# A New View of the Lethal Apoptotic Pore

**DOI:** 10.1371/journal.pbio.1001399

**Published:** 2012-09-25

**Authors:** Gorka Basañez, Lucian Soane, J. Marie Hardwick

**Affiliations:** 1 Biophysics Unit, Spanish Science Research Council (CSIC) and University of the Basque Country (UPV/EHU), Bilbao, Spain; 2 Department of Molecular Microbiology and Immunology, Johns Hopkins School of Public Health, Baltimore, Maryland, United States of America

## Abstract

Cell death by apoptosis is indispensable for proper development and tissue homeostasis in all multicellular organisms, and its deregulation plays a key role in cancer and many other diseases. A crucial event in apoptosis is the formation of protein-permeable pores in the outer mitochondrial membrane that release cytochrome *c* and other apoptosis-promoting factors into the cytosol. Research efforts over the past two decades have established that apoptotic pores require BCL-2 family proteins, with the proapoptotic BAX-type proteins being direct effectors of pore formation. Accumulating evidence indicates that other cellular components also cooperate with BCL-2 family members to regulate the apoptotic pore. Despite this knowledge, the molecular pathway leading to apoptotic pore formation at the outer mitochondrial membrane and the precise nature of this outer membrane pore remain enigmatic. In this issue of *PLOS Biology*, Kushnareva and colleagues describe a novel kinetic analysis of the dynamics of BAX-dependent apoptotic pore formation recapitulated in native mitochondrial outer membranes. Their study reveals the existence of a hitherto unknown outer mitochondrial membrane factor that is critical for BAX-mediated apoptotic pore formation, and challenges the currently popular view that the apoptotic pore is a purely proteinaceous multimeric assembly of BAX proteins. It also supports the notion that membrane remodeling events are implicated in the formation of a lipid-containing apoptotic pore.

Apoptosis is the orderly sequence of events that leads to the death of a cell without releasing harmful substances into the surrounding tissue; it is indispensable for normal embryonic development and maintenance of healthy tissues in all multicellular organisms and important in many pathologies. The death of neurons and lymphocytes by apoptosis, for example, contributes to neurodegeneration and AIDS, respectively. By ensuring the death of damaged cells, apoptosis also plays key roles in cancer prevention and in successful cancer treatment. Over 25 years of apoptosis research have led to the broadly accepted notion that mitochondria, traditionally viewed as the “powerhouses” of the cell, are also intimately linked to cell death.

Apoptosis can be initiated either by the activation of cell-surface-expressed death receptors or by diverse intracellular signals that impinge on the mitochondria. In vertebrates, the commitment step in the mitochondrial pathway of apoptosis is the assembly of a supramolecular structure called the apoptotic pore in the outer mitochondrial membrane [1]. This outer membrane pore allows for rapid diffusion out of the mitochondria of cytochrome *c* and other proteins that promote the irreversible dismantling of the cell. Despite intense research efforts, our understanding of the molecular machinery and mechanisms implicated in this crucial aspect of apoptosis is still incomplete.

## BCL-2 Family Proteins Regulate the Apoptotic Pore

BCL-2 family proteins have long been considered the main regulators of the mitochondrial pathway of apoptosis, and have also been found recently to have additional roles in other physiological and pathological settings [2]. The important role of BCL-2 family proteins in cell fate decisions is highlighted by the fact that compounds that target BCL-2 family proteins are advancing in clinical trials as potential next-generation therapeutics against cancer and degenerative diseases [3]. BCL-2 family members can be subdivided into three functional groups: (1) BCL-2-type anti-apoptotic proteins (BCL-2, BCL-X_L_, and others), which inhibit apoptotic pore formation; (2) pro-apoptotic BAX-type proteins (BAX and BAK), which, once activated, act as direct effectors of the apoptotic pore, and (3) pro-apoptotic BH3-only proteins (such as BID and BIM), which trigger functional BAX/BAK activation [4].

Solution studies have revealed the potential of specific pro-apoptotic and anti-apoptotic BCL-2 family members to engage in selective protein–protein interactions; however, exactly how the three factions of the BCL-2 family perform their duties at the outer mitochondrial membrane remains a major question for apoptosis researchers. In particular, accumulating evidence indicates that insertion into the outer mitochondrial membrane causes functionally important conformational changes in BCL-2 family proteins [5]. Another important question that has recently attracted considerable attention is the possibility that cellular factors besides BCL-2 family proteins are also involved in forming the apoptotic pore. Specifically, many observations indicate a close functional relationship between the BCL-2 family and the mitochondrial fission and fusion machinery during apoptosis [6]. Currently, the mechanistic basis of this relationship is hotly debated, which is perhaps not surprising given that both BCL-2 family proteins and mitochondrial fission and fusion proteins appear to have multiple functions [2],[6]. On top of this, recent studies indicate that the location of several BCL-2 family proteins in mitochondria is more complex and dynamic than previously anticipated [7]–[9]. The multilayered biochemical, structural, and dynamic complexities of intact cells undergoing cell death are challenging to dissect, leading many investigators to study simpler, more well-defined model systems.

Model systems reconstituted in vitro from purified components provide a powerful experimental means to obtain mechanistic information about biological processes. In apoptosis research, studies with recombinant proteins and pure lipid vesicles (liposomes) were instrumental in establishing that BAX-type proteins have intrinsic pore-forming activity. In the absence of additional mitochondrial factors, the pore-forming function of BAX and BAK can be activated by selected BH3-only proteins or peptides containing their BH3 domains [10]–[13]. Studies with the antiapoptotic BCL-X_L_ protein reconstituted in liposomes provided important mechanistic insights into how it inhibits BAX activity [14],[15], and how a cleaved form of BCL-X_L_ with proapoptotic function acquires a BAX-like pore-forming activity [16],[17]. Moreover, liposome studies led to the currently popular model in which functional BAX activation proceeds through an ordered series of events consisting of BAX translocation to the membrane triggered by BH3-only proteins, extensive integration of BAX into the membrane, and oligomerization to form the apoptotic pore [18].

## Two Models for Apoptotic Pore Formation

In this issue of *PLOS Biology*, Donald Newmeyer, Yulia Kushnareva, and colleagues studied the kinetics of BAX-dependent pore formation recapitulated in isolated mitochondrial outer membrane vesicles (OMVs) [19]. This novel cell-free experimental approach revealed a number of unexpected and important aspects of the molecular pathway leading to apoptotic pore formation. Foremost, the authors found that the kinetics of membrane permeabilization triggered by BAX in OMVs were noticeably more complex than the kinetics in liposomes: OMV permeabilization had a pronounced early latency phase that was absent in liposomes, which lack other cellular factors. Importantly, as observed with isolated OMVs, intact mitochondria treated with activated BAX also displayed an early latency phase in their permeabilization kinetics. Based on these and other observations, the authors concluded that the BAX-induced liposome permeabilization process only partially reproduces the BAX-dependent route for membrane permeabilization in the context of native outer mitochondrial membranes. Thus, the latency phase represents a new and experimentally tractable point of regulation in the mitochondrial apoptosis pathway.

So, what is the origin of the striking latency phase observed in the BAX-dependent OMV permeabilization process? By using several biochemical and biophysical approaches the Newmeyer team demonstrated that this latency phase reflects neither the inhibition of BAX activation by BCL-2-type proteins at the outer mitochondrial membrane, nor the accumulation of a threshold amount of membrane-integrated BAX. The authors went on to build a robust mathematical model that accurately fits the entire kinetic response, consisting of two distinct but coupled reactions: the first reaction during the latency phase represents the assembly of a multimeric catalyst that is required for the second, pore formation reaction. Remarkably, further experimentation and theoretical analysis revealed that the multimeric catalyst is not BAX itself, as might have been predicted from previous knowledge. Furthermore, the observed kinetics also imply that BAX oligomerization is irrelevant for the reaction scheme. This is another surprising and important contribution of the study because, as alluded to above, it challenges the commonly held assumption that the apoptotic pore consists of multimeric BAX (or BAK) proteins. Rather, the data favor another scenario, where the active agents of apoptotic pore formation are membrane-integrated BAX monomers assisted by a multimeric non-BAX catalyst complex.

Although intuitively appealing, it is currently uncertain whether assembly of higher-order oligomeric BAX (or BAK) complexes at the outer mitochondrial membrane is causally linked to formation of apoptotic pores, or merely accompanies apoptosis as an epiphenomenon. One argument for a causal role is evidence showing that BAX-channel inhibitors block cytochrome *c* release [20], although the mechanism of action of these inhibitors has not been validated thoroughly. Unfortunately, structural information about higher-order multimeric species of BAX-type proteins that might constitute the functional apoptotic pore is currently scarce [21], although atomic-resolution structural data are available for several BCL-2 family members (including BAK) in their dimeric forms [22]–[25].

An alternative explanation for the kinetics data, favored by Newmeyer and colleagues, is the lipidic pore model [10],[13],[26]–[28]. In this model, the lumen of the pore is lined, at least partially, by lipid molecules. Mechanistically, insertion of amphipathic segments of BAX-type proteins into the outer leaflet of the membrane (without fully traversing the membrane) is proposed to destabilize the membrane bilayer structure, inducing lipids to fold into a highly curved pore. Interestingly, lipidic pores that are implicated in membrane fusion are purportedly caused by fusogenic proteins arranged in a ring-like manner to isolate a local piece of a membrane [29], but protein oligomerization is not strictly required for lipidic pore formation. In fact, many amphipathic antimicrobial peptides and bacteriocins that are structurally related to BAX/BAK (e.g., the colicins and diphtheria toxin) have been proposed to permeabilize membranes by forming lipidic pores while retaining a monomeric structure [30]–[32]. Whether the apoptotic pore is a lipid-containing pore or a exclusively proteinaceous pore, elucidating its exact nature will remain a formidable challenge for the coming years, which will likely require not only a combination of conventional biophysical and theoretical approaches, but also information gathered with emerging technologies.

## The Missing Factor for Apoptotic Pore Formation

An obvious question raised by the Newmeyer team's study is the identity of the multimeric catalyst in the outer mitochondrial membrane that stimulates BAX-dependent pore formation. One important clue obtained from this study is that this “missing factor” possesses membrane-remodeling activity. Considering the experimental link between multimeric catalyst assembly and a membrane phase-transition like event, together with the evidence that membrane-active components of the mitochondrial fission/fusion machinery are involved in apoptosis regulation, several candidates come to mind. Two likely candidates are DRP1, a conserved dynamin-like GTPase required for mitochondrial fission [6], and BIF-1/SH3GLB1/endophilin-B1, a BAX-binding protein also involved in mitochondrial shape changes [33]. DRP1 and BIF-1 each form multimeric complexes with membrane-remodeling activities, they can both cooperate with BAX to permeabilize the outer mitochondrial membrane during apoptosis, and some of these effects have been reconstituted in liposomes [6],[33]–[36]. Kushnareva and colleagues clearly show that the “missing factor” is not a canonical (nucleotide-dependent) DRP1 activity and provide evidence against BIF-1 implication. However, their data do not rigorously exclude participation of DRP1 or BIF-1.

Other prime candidates for the missing factor are the mitofusins MFN1 and MFN2, two outer mitochondrial membrane proteins with a well-established role in mitochondrial fusion (a process that also involves membrane remodeling), and with the potential to form multimeric complexes [34]. Mitofusins were recently recognized as functional partners of BAX-type proteins under normal conditions in healthy cells and also during certain types of cell death [34],[37]. MFN2 has also been implicated in tethering mitochondria to the endoplasmic reticulum (ER) in combination with other protein complexes [38],[39], and we note that the isolated OMV system employed in the Newmeyer team's study was substantially contaminated with ER. Based on these observations, together with the recent discovery that certain ER-derived sphingolipid metabolites play a key role in BAX/BAK activation and action [40], the missing factor that stimulates BAX pore formation may well be a lipid-degrading enzyme located in the ER or in the outer mitochondrial membrane, or it may even be a lipid rather than a protein. Whether the specific BAX-activating sphingolipid metabolites display membrane-remodeling activity is unknown. Another mitochondrial lipid that deserves mention in this context is cardiolipin, which also has the potential to remodel mitochondrial membrane structure, in addition to its ability to interact functionally with BAX, BAK, and mitochondrial fission/fusion proteins [10],[13],[35],[36],[41]. Nevertheless, the authors favor a proteinaceous factor and do not favor a requirement for cardiolipin in the kinetic scheme of BAX-dependent pore formation recapitulated in OMVs. Finally, the missing factor might be a molecule involved in mitochondrial dynamics and apoptosis that has not yet been identified.

**Figure 1:**
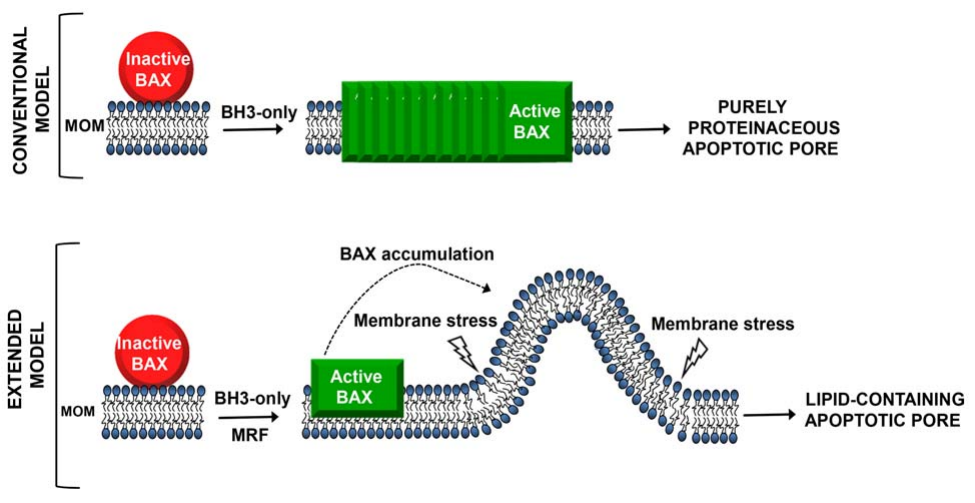
Two models for formation of the apoptotic pore in the outer mitochondrial membrane. In the conventional model, proapoptotic BCL-2 family members are the only molecules implicated in pore formation, with BH3-only proteins promoting conversion of BAX from an inactive monomeric state to form a multimeric BAX channel structure that acts as purely proteinaceous apoptotic pore. The extended model, supported by Newmeyer and colleagues, proposes that the apoptotic pore is regulated by the concerted action of proapoptotic BCL-2 family members together with membrane-remodeling factors (MRF) in the outer mitochondrial membrane. In this model, BH3-only proteins act in concert with MRF to promote the BAX-driven formation of a lipid-containing apoptotic pore. MRF might operate by generating membrane curvature, which could lead to BAX accumulation at this specific membrane microdomain, and/or could cause membrane stress that facilitates formation of a lipidic pore. MRF may be one or more proteins, but could potentially be a lipid, or a combination of proteins and lipids.

Whatever the identity of the missing factor, another significant remaining challenge is to elucidate the precise manner by which it stimulates BAX-dependent apoptotic pore formation. One mechanism that can be envisaged based on our current knowledge of membrane remodeling is induction of membrane curvature. Both proteins and lipids can generate membrane curvature by a variety of mechanisms [42]. Selective recruitment of BAX to highly curved outer mitochondrial membrane microdomains generated by the missing factor might lead to increased local concentration of BAX in the membrane (Figure 1). Alternatively, or in addition, increased membrane curvature could also produce membrane stress that directly favors formation of the aperture and/or expansion of a lipid-containing pore. Indeed, shallow membrane insertion of amphipathic helices or hydrophobic loops as curvature-effecting protein modules that disturb membrane integrity is an emerging theme in membrane fission [43]. It is equally possible, of course, that the missing factor may act by a mechanism unrelated to curvature generation, such as lateral segregation of membrane lipids. Without doubt, these will be exciting avenues for future basic research. They also hold promise from a pharmacological viewpoint since each new step and molecule identified in the molecular pathway leading to apoptotic pore formation provides a potential new therapeutic target.

## Abbreviations

ER: endoplasmic reticulum
MRF: membrane-remodeling factors
OMV: outer membrane vesicle
